# Accuracy of cartilage-specific 3-Tesla 3D-DESS magnetic resonance imaging in the diagnosis of chondral lesions: comparison with knee arthroscopy

**DOI:** 10.1186/s13018-015-0326-1

**Published:** 2015-12-29

**Authors:** Sandro Kohl, Simon Meier, Sufian S. Ahmad, Harald Bonel, Aristomenis K. Exadaktylos, Anna Krismer, Dimitrios Stergios Evangelopoulos

**Affiliations:** Department of Orthopaedic Surgery, Inselspital, University of Bern, Freiburgstrasse, CH-3010 Bern, Switzerland; Department of Diagnostic, Interventional and Pediatric Radiology, Inselspital, University of Bern, Freiburgstrasse, Bern, CH-3010 Switzerland; Department of Emergency Medicine, Inselspital, University of Bern, Freiburgstrasse, Bern, CH-3010 Switzerland; 3rd Department of Orthopaedic Surgery, KAT Hospital, University of Athens, Athens, Greece

**Keywords:** Knee arthroscopy, Cartilage lesion, 3-Tesla MRI, 3D-DESS

## Abstract

**Background:**

Arthroscopy is considered as “the gold standard” for the diagnosis of traumatic intraarticular knee lesions. However, recent developments in magnetic resonance imaging (MRI) now offer good opportunities for the indirect assessment of the integrity and structural changes of the knee articular cartilage. The study was to investigate whether cartilage-specific sequences on a 3-Tesla MRI provide accurate assessment for the detection of cartilage defects.

**Methods:**

A 3-Tesla (3-T) MRI combined with three-dimensional double-echo steady-state (3D-DESS) cartilage specific sequences was performed on 210 patients with knee pain prior to knee arthroscopy. Sensitivity, specificity, and positive and negative predictive values of magnetic resonance imaging were calculated and correlated to the arthroscopic findings of cartilaginous lesions. Lesions were classified using the modified Outerbridge classification.

**Results:**

For the 210 patients (1260 cartilage surfaces: patella, trochlea, medial femoral condyle, medial tibia, lateral femoral condyle, lateral tibia) evaluated, the sensitivities, specificities, positive predictive values, and negative predictive values of 3-T MRI were 83.3, 99.8, 84.4, and 99.8 %, respectively, for the detection of grade IV lesions; 74.1, 99.6, 85.2, and 99.3 %, respectively, for grade III lesions; 67.9, 99.2, 76.6, and 98.2 %, respectively, for grade II lesions; and 8.8, 99.5, 80, and 92 %, respectively, for grade I lesions.

**Conclusions:**

For grade III and IV lesions, 3-T MRI combined with 3D-DESS cartilage-specific sequences represents an accurate diagnostic tool. For grade II lesions, the technique demonstrates moderate sensitivity, while for grade I lesions, the sensitivity is limited to provide reliable diagnosis compared to knee arthroscopy.

## Background

Focal cartilage defects display poor healing ability and favor the development of early osteoarthritis [1]. Arthroscopy is considered as “the gold standard” for the diagnosis of traumatic intraarticular knee lesions, having accuracy as high as 95 to 98 % [2–5]. This technique, however, is invasive and expensive and displays potential complications [6, 7]. Recent developments of ultra-high-field magnetic resonance imaging (MRI) offer another method for indirect assessment of the integrity and structural changes of the articular cartilage of the knee joint. [8].

Several recent studies report on the sensitivity and specificity of the MRI in detecting meniscal and ligamentous lesions of the knee [9, 10]. However, cartilage lesions are difficult to evaluate by standard MRI, even when cartilage-specific sequences are applied [11–14]. Recent data support that the addition of a T2 mapping sequence to a routine MR protocol at 3.0 T improved sensitivity in the early detection of cartilage degenerative lesions [15]. Still, 3D dual echo in the steady state (DESS) sequences have been shown to provide improved universal cartilage discrimination with sufficient retest precision [16, 17]. Three 3-Tesla (3-T) MRI displays a distinct signal gain that may be transferred into a higher spatial resolution as well as a shorter acquisition time, thus facilitating the detection of cartilaginous lesions [11, 18]. Crema et al., applying 3D spoiled gradient recalled echo (SPGR) sequences (for cartilage thickness) and 3D inversion recovery-prepared SPGR sequences after delayed gadolinium-enhanced MRI of cartilage (dGEMRIC), reported an association between decrease in dGEMRIC indices and early stages of cartilage degeneration [19]. Van Dyck et al. stated that cartilage imaging of the knee at 3 T can be reliably performed using 3D-TSE, showing high accuracy when compared to standard sequences [20].

Literature data support a uniform (medial, lateral) distribution of grade I/II defects in 3-T MRI exam. High-grade lesions (grade III/IV), however, showed a predilection for medial localization [21–26]. Intra-articular knee lesions are associated with significant morbidity and frequently require surgical treatment. The purpose of the study was to investigate whether cartilage-specific sequences on a 3-Tesla MRI provide accurate assessment for the detection of cartilage defects. Authors’ hypothesis was that 3-T MRI combined with cartilage-specific three-dimensional double-echo steady-state (3D-DESS) sequences represents an accurate method for the detection of cartilage defects in comparison with arthroscopy, as the standard and that high-grade cartilaginous lesions (grade III/IV) demonstrate a predilection for medial localization.

## Methods

For 210 patients with knee pain, 3-T MR imaging including cartilage-specific 3D-DESS sequences was prospectively performed prior to knee arthroscopy. All patients were sampled consecutively from the outpatient clinic of the authors’ institution (University Orthopaedic Department, level A Trauma Centre) and were submitted to anteroposterior and lateral knee X-rays prior to MR imaging. All demonstrated cartilaginous lesions and composed the study group. Inclusion criteria were a history of knee pain greater than 3 months and clinical suspicion of internal knee pathology. Patients with a history of ipsilateral knee surgery, prior intra-articular fractures, evidence of ligamentous injury, advanced osteoarthritis, or with externally obtained MRI were excluded from the study. This study was approved by the Inselspital review board and ethics committee. All patients provided written informed consent.

### MRI protocol

All MRI examinations were performed with an advanced 3-Tesla MRI scanner (Magnetom Verio TIM, software version VB 17, Siemens, Erlangen, Germany) using a dedicated 15-channel phased array knee coil. Knee joint cartilage was evaluated using standard MRI sequences as well as an isotropic multiplanar reconstructible 3D-DESS sequence (dual echo steady state gradient recalled echo) with selective water excitation (WE). The repetition time (TR) was 5 ms, and the echo time (TE) was 14.2 ms. The flip angle (FA) was 25°. An isotropic voxel size with a volume of 0.33 mm^3^ was applied for cartilage imaging [27]. For localization of the cartilage lesions, multiplanar reconstructions in axial, sagittal, and coronal planes optimized for the anatomy of the joint were used. Cartilage lesions were classified based on their extent and depth using the modified Outerbridge classification [28–32].

MR images were reviewed separately by two independent readers, an orthopedic surgeon experienced on musculoskeletal imaging and a radiologist. Both were blinded to clinical data, including surgical reports. Interclass correlation coefficient (ICC) was calculated among the two independent readers for each grade of lesions identified on MRI evaluations [33].

To compare the MRI results to those found at arthroscopy, the articular surface of the knee was divided into six regions: patella, trochlea, medial femoral condyle, medial tibia, lateral femoral condyle, and lateral tibia. Areas were consecutively marked with an “M” and “L,” respectively. For each femoral, tibial, trochlear, and patellar region, the occurrence of grades I–IV Outerbridge lesion at each position was tabulated by frequency tables. Each cartilage surface was analyzed as a single entity. To perform a direct comparison between MRI and arthroscopy, we used a classification based on the Outerbridge macroscopic grading [30, 32]. Grade 0 is defined as cartilage with a normal intrinsic signal and surface contour while signal heterogeneities within the cartilage in the presence of a smooth surface were rated as grade I lesions and conform to the arthroscopic finding of a cartilage softening. Grade 2 were defined as the lesions showing fibrillation or erosion composing less than 50 % of the cartilage thickness while defects of more than 50 % with or without small bone ulcerations were defined as grade III. Full-thickness cartilage lesions were defined as grade IV. In cases of multiple cartilage defects within one of the six articular surfaces, only the highest grade of cartilage damage was documented.

### Arthroscopy

Indications for arthroscopic surgery included documentation and treatment of cartilage and meniscal lesions as well as joint instability. Arthroscopic grading of cartilage disorders was performed by an orthopedic surgeons experienced in knee surgery. MRI images were available to the surgeon prior to knee arthroscopy, whereas the MRI grading of the hyaline cartilage was not present. Arthroscopies were performed through standard anteromedial and anterolateral portals. Arthroscopic findings were classified in grades 0 to IV according to the system of Outerbridge [8, 30]. Cartilage lesions were recorded in a standardized documentation sheet derived from the mapping method employed by the International Cartilage Repair Society (ICRS) [34]. All arthroscopies were performed by the same surgeon within 2 weeks from MRI.

### Statistical analysis

Statistical analysis was performed using the Statistical Package for the Social Sciences (SPSS) version 12. Comparisons between qualitative variables were performed using the chi-square test. *p* values <0.05 were considered as statistically significant. Sensitivity, specificity, and positive and negative predictive values, *p* values, and kappa agreement measures were calculated to test the validity of MRI compared against arthroscopy.

## Results

The study included 210 patients, all exhibiting cartilage defects (1260 articular surfaces). Of those, 90 were women and 120 were men with a mean age of 42 years (range 18–81 years). The total number of detected lesions on arthroscopy and MRI for each anatomical location (femur, tibia, patella, and trochlea) is illustrated on Table 1. A total of 473 lesions were arthroscopically detected: 248 (52.4 %) were grade I lesions, 131 (27.7 %) grade II, 63 (13.3 %) grade III, and 31 (6.6 %) grade IV. The area most commonly affected was the patellar compartment with 99 (20.9 %) lesions, followed by the medial tibial plateau with 92 (19.5 %), the medial femoral condyle with 85 (18.0 %), the lateral femoral condyle with 71 (15.0 %), the lateral tibial plateau with 68 (14.4.0 %), and the trochlea with 58 (12.2 %) lesions.

**Table 1:** Distribution of the cartilaginous lesions (arthroscopy/MRI)

Grade	Femur	Tibia	Patella	Trochlea
GI	84/9	80/8	49/17	35/4
GII	38/26	53/30	27/24	13/10
GIII	23/16	19/12	19/18	3/3
GIV	13/13	8/7	4/4	5/5

**Fig. 1:**
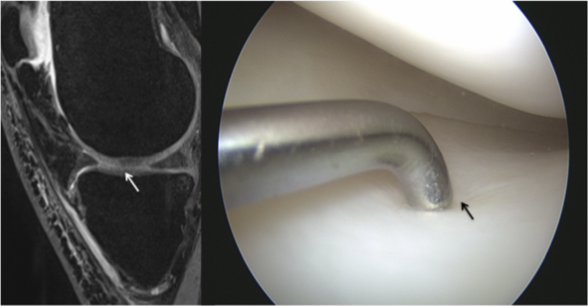
MRI and arthroscopic image of a grade I lesion in an 18-year-old male. The lesion is hardly visible in sagittal source images, coronal reconstructions with a slice thickness of 1.5 mm already miss the grade I lesion

**Fig. 2:**
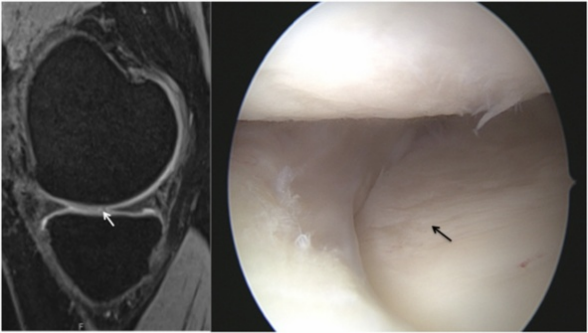
MRI and arthroscopic image of a grade II lesion in a 32-year-old male patient, reported by both readers. It is well visible in the sagittal images

**Fig. 3:**
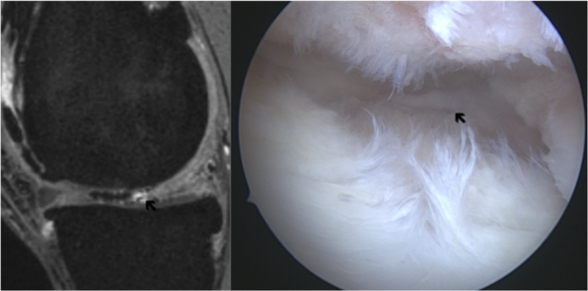
MRI and arthroscopic image of a grade III lesion in a 42-year-old male patient. The lesion is hard to miss. The cartilage remnants can be well seen and therefore a grade IV lesion can be ruled out

**Fig. 4:**
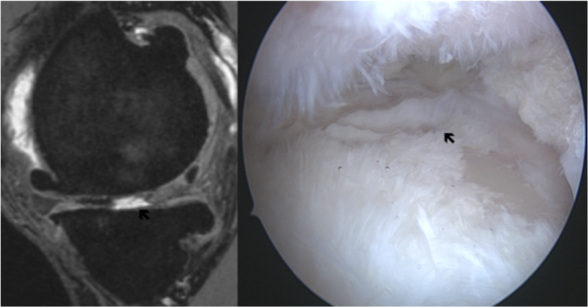
MRI and arthroscopic image of a grade IV lesion in a 42-year-old patient completely depleted of cartilage in the weight bearing area

MRI detected a total of 202 lesions. Of those, 38 (18.8 %) were grade I, 90 (44.5 %) grade II, 45 (22.3 %) grade III, and 29 (14.4 %) grade IV. The area most commonly affected was the patellar compartment with 61 (30.1 %) lesions, followed by the medial femoral condyle with 41 (20.3 %), the medial tibial condyle with 38 (18.8 %), the lateral femoral condyle with 24 (11.9 %), the trochlea with 20 (9.9 %), and the lateral tibial condyle with 18 (8.9 %) lesions (Figs. 1, 2, 3, and 4). ICC for each grade of lesions identified on MRI evaluations was 0.34 (*p* = 0.03) for grade I, 0.67 (*p* < 0.001) for grade II, 0.89 (*p* < 0.001) for grade III, and 0.99 (*p* < 0.001) for grade IV lesions, showing consistency between the two independent reviewers for grades II–IV lesions.

Statistical analysis reporting sensitivity, specificity, positive predicting value, and negative predicting value according to the grade and the localization of the lesions is shown on Table 2. Data show that 3D-DESS 3-T MRI displayed low sensitivity and high specificity in the detection of grade I cartilaginous lesions in various knee compartments. However, for grade II–IV lesions, higher values of sensitivity were documented. The highest sensitivity was noted at the patellar compartment of grade IV lesion while the lowest at the femoral condyle of grade I lesions.

**Table 2:** Topographic statistical analysis

Outerbridge grade	Sensitivity	Specificity	Positive predictive value	Negative predictive value	*p* value
I	8.8	99.5	80.0	92.0	0.00
II	67.9	99.2	76.6	98.2	0.00
III	74.0	99.6	85.2	99.3	0.00
IV	83.3	99.8	84.4	99.8	0.00

## Discussion

Focal cartilage defects favor the development of early knee osteoarthritis. Although various classifications of cartilaginous changes of the knee have been applied in the current literature, the modified Outerbridge classification remains the descriptive standard for macroscopic evaluation of the cartilage [1, 28–31].

MRI represents the best imaging technique currently available for the assessment of articular cartilage displaying an excellent soft-tissue contrast [34, 35]. It has the capacity of providing morphologic information on cartilage defects, such as fissuring and partial- or full-thickness lesions. Non-invasive procedures such as MRI are being vigorously discussed regarding their sensitivity and specificity and have so far produced good results. Literature data reports that MRI represents a sensitive and accurate tool for evaluating the morphology and structure of joint cartilage, as well as the accompanying pathological changes [36–38]. It has been shown that 3-Tesla equipments have become very efficient in showing the whole articular cartilage and classifying the degenerative damage by analyzing morphological, structural, and physical properties. Whether or not MRI underestimates the number and size of cartilage lesions of the knee joint is a point of controversy [36]. There is general agreement that cartilage damage evaluation on standard MRI examination remains problematic. However, recently developed cartilage-specific MRI sequences were able to demonstrate, depending on the severity of the lesion, a sensitivity of 43 to 87 % and a specificity of 89 to 97 % [21, 39–42]. In the present study, joint cartilage was examined using the well-established 3D-DESS sequence on a 3-Tesla MRI scanner, which various studies have shown to be very well suited for this purpose and therefore has served as a standard of reference in cartilage imaging in many major preceding studies [37–39]. In addition to visualizing the actual cartilage thickness plus adequate contrasting of the cartilage-bone border, this sequence has the advantage of enabling multiplanar three-dimensional reconstruction, thus allowing detailed evaluation of joint cartilage lesions [16, 43, 44].

The results of this study show that the highest sensitivity values were encountered at the articular surface of the patella for all grades of lesions (sensitivity range from 26.0 % for grade I to 100.0 %for grade IV lesions). However, further statistical analysis incorporating the grade of the cartilaginous defects and the modified Outerbridge classification demonstrated a significant low sensitivity and high specificity for grade I lesions while higher sensitivity values were encountered for grade II–IV lesions, respectively (Table 2).

Similar studies report sensitivities ranging from 14 to 100 % and specificities ranging from 80 to 100 % for the detection of partial- and full-thickness cartilage lesions [45–47]. Using a 3-T MRI and the cartilage-specific 3D-DESS sequences, our diagnostic values for the detection of grade III and IV lesions were well within these ranges. In addition, for the obscure grade II lesions, 3-T MRI showed a relatively good sensitivity (mean sensitivity 67.9 %, range 55.8 to 73.8 %) compared with the literature, where grade II lesions either are not detectable or show sensitivities ranging from 14 to 67 %. [14, 45–48].

The results of this study suggest that the value of 3-T MRI, even when combined with cartilage-specific 3D-DESS sequences, still remains limited for the detection of grade I changes. Similarly, Potter et al. reported that in some cases, MR images suggested grade I lesions that could not be verified by subsequent arthroscopy [49]. The data demonstrate that 3D-DESS MRI, although very efficient in detecting deep cartilage defects, may provide moderate accuracy for grade II lesions. For grade I lesions, however, the obtained accuracy remains limited compared to minimal invasive techniques such as arthroscopy [14, 37, 48–51]. Recent data on the addition of T2 mapping sequence to a routine MR protocol at 3.0 T report improved sensitivity in the early detection of cartilage degenerative lesions [15]. Moreover, the application of 3D SPGR (for cartilage thickness) and 3D inversion recovery-prepared SPGR sequences after dGEMRIC, has demonstrated good results in the diagnosis of early-stage cartilage degeneration [19].

von Engelhardt et al. reported a uniform (medial, lateral) distribution of grade I/II defects in 3-T MRI exams [40]. High-grade lesions (grade III/IV), however, showed a predilection for medial localization. Their results correspond closely with those of the present study. Overall, 29.8 % of the detected lesions were located in the lateral compartment and 37.2 % in the medial compartment. In our cohort, higher-grade lesions were located in the medial knee joint compartment. Several studies report cartilaginous lesions occurring preferentially in the medial compartment and/or in the medial femoral condyle [1, 22–26, 42].

### Limitations

Despite our efforts to ensure the validity of the present study, certain limitations are observed. The size of this patient cohort is limited to allow permanent conclusions. A further limitation represents the lack of comparative group with 1.5-T MR. Therefore, no comparisons could be performed between 3- and 1.5-T MR images. Additionally, our radiological evaluation included only standard as well as 3D-DESS sequences on a 3-T MRI. At that time, these were the sequences used for cartilage investigation at our institution. DESS has been widely applied for cartilage evaluation [42]. 3D sequences have been proposed by the Osteoarthritis Initiative to provide additional coronal and axial reconstructions for improved cartilage evaluation [16]. However, nowadays, intermediate (fat saturated) FSE/TSE as well as 3D SPGR sequences (for cartilage thickness) and 3D inversion recovery-prepared SPGR sequences after dGEMRIC sequences have also been applied, providing superior cartilage imaging, even for the early lesions [19, 52, 53]. In a recent review of Sasho, the author suggests two types of MR imaging for the evaluation of knee cartilage: Whole-Organ Magnetic Resonance Imaging Score (WORMS) for the assessment of the status of knee joint as a whole as well as specific sequences such as dGEMRIC, T2 mapping, and T1 rho for the assessment of cartilage status [54].

## Conclusions

The results of this study demonstrate that 3-T MRI with conventional and 3D-DESS cartilage-specific sequences represents an accurate diagnostic tool for grade III and IV lesions. Moderate sensitivity was encountered for grade I and II lesions; the technique displays limited sensitivity to provide reliable diagnosis compared to knee arthroscopy for early cartilage defects.
